# Clinical response to belimumab in academic clinical practices

**DOI:** 10.1186/ar4665

**Published:** 2014-09-18

**Authors:** Arthi Reddy, XiaoQing Li, Jill P Buyon, Andrew G Franks, Richard A Furie, Diane L Kamen, Susan Manzi, Michelle Petri, Rosalind Ramsey-Goldman, Chung-E Tseng, Ronald F van Vollenhoven, Daniel J Wallace, Anca D Askanase

**Affiliations:** 1 New York University Langone Medical Center, New York, NY, USA; 2 Columbia University Medical Center, New York, NY, USA; 3 Hofstra North Shore-Long Island Jewish School of Medicine, Great Neck, NY, USA; 4 Medical University of South Carolina, Charleston, SC, USA; 5 Temple University School of Medicine, Philadelphia, PA, USA; 6 Johns Hopkins University School of Medicine, Baltimore, MD, USA; 7 Northwestern University Feinberg School of Medicine, Chicago, IL, USA; 8 Karolinska University Hospital, Stockholm, Sweden; 9 Cedars-Sinai Medical Center, Los Angeles, CA, USA

## Background

Belimumab is a human monoclonal antibody that inhibits soluble B-lymphocyte stimulator and improves systemic lupus erythematosus (SLE) disease activity. This study was initiated to evaluate the use and efficacy of belimumab in academic SLE clinical practices.

## Methods

An invitation to participate was sent to 16 physicians experienced in SLE phase III clinical trials. All agreeing to participate completed a one-page questionnaire for each patient prescribed belimumab that includes demographic and SLE characteristics, and information about belimumab administration. The questionnaire completed every 3 months by the physicians also captured clinical responses and belimumab safety. Clinical response was defined as a ≥50% improvement in the initial clinical manifestation being treated without worsening in other organ systems.

## Results

Of 16 invitations sent, nine investigators participated. Questionnaires on 150 patients treated with belimumab for at least 3 months were available for analysis. The mean age was 41.9 ± 12.6 years. 92.0% were female, 67.1% White, 24.7% Black, 5.7% Asian, and 5.3% Hispanic. The average SLE disease duration was 12.2 ± 8.2 years. Concomitant medications included: prednisone in 73.3% (mean dose of 12.2 ± 10.9, 41.7% on ≥10 mg), antimalarials in 71.7%, and immunosuppressants in 66.3% (mycophenolate mofetil 34.2%, azathioprine 20.3%, methotrexate 11.8%). Only 3.7% of patients were not on any background SLE medications, 8.0% were on antimalarials alone. The dominant clinical manifestations driving treatment were arthritis in 69.5%, rash in 44.4%, and inability to taper steroids in 27.3%. Other SLE manifestations were serositis 16.0%, hematological 13.9%, and renal 10.7%. A total 65.2% of patients had ≥2 active manifestations. Of the 150 patients on belimumab for at least 3 months, 69 (46.0%) clinically responded by 3 months with marked improvement in arthritis and/or rash. Similarly, of the 112 patients on belimumab for at least 6 months for whom follow-up data were available, 54 (48.2%) clinically responded with improvements in arthritis, rash and/or nephritis. While the numbers are limited, black patients showed improvement at 6 months, with 19/26 (73%) of patients responding, *P *= 0.05.

## Conclusions

These observational data support the use of belimumab across all racial/ethnic groups and efficacy similar to that reported in the phase III trials. Relevant to physician and patient decision-making, improvement was seen as early as 3 months.

